# Supplementary material for: Modeling glioblastoma heterogeneity as a dynamic network of cell states
Source: Mol Syst Biol. 2021 Sep 16;17(9):e10105. doi: 10.15252/msb.202010105 (PMC8444284; doi:10.15252/msb.202010105)
Supplement: Supplementary file 6 — Source Data for Figure 5 [file MSB-17-e10105-s004.zip › Figure5A_sourcedata/GSEA_3017/hallmarks_stateC.GseaPreranked.1621934520804/HALLMARK_COMPLEMENT.html]

Details for gene set HALLMARK\_COMPLEMENT[GSEA]

|  || Dataset | state13017 |
| Phenotype | NoPhenotypeAvailable |
| Upregulated in class | na\_neg |
| GeneSet | HALLMARK\_COMPLEMENT |
| Enrichment Score (ES) | -0.51149875 |
| Normalized Enrichment Score (NES) | -1.958037 |
| Nominal p-value | 0.0031897926 |
| FDR q-value | 0.00830997 |
| FWER p-Value | 0.046 |
Table: GSEA Results Summary

  

Fig 1: Enrichment plot: HALLMARK\_COMPLEMENT      
 Profile of the Running ES Score & Positions of GeneSet Members on the Rank Ordered List

  

| PROBE | GENE SYMBOL | GENE\_TITLE | RANK IN GENE LIST | RANK METRIC SCORE | RUNNING ES | CORE ENRICHMENT || 1 | ATOX1 |  |  | 229 | -0.260 | -0.3517 | No |
| 2 | SERPINE1 |  |  | 323 | -0.286 | -0.4656 | Yes |
| 3 | GNB4 |  |  | 335 | -0.289 | -0.4380 | Yes |
| 4 | COL4A2 |  |  | 353 | -0.300 | -0.4189 | Yes |
| 5 | LRP1 |  |  | 366 | -0.304 | -0.3907 | Yes |
| 6 | S100A13 |  |  | 396 | -0.319 | -0.3892 | Yes |
| 7 | TIMP1 |  |  | 399 | -0.320 | -0.3411 | Yes |
| 8 | HSPA5 |  |  | 447 | -0.350 | -0.3655 | Yes |
| 9 | LGALS3 |  |  | 471 | -0.374 | -0.3448 | Yes |
| 10 | F3 |  |  | 505 | -0.432 | -0.3320 | Yes |
| 11 | CA2 |  |  | 518 | -0.448 | -0.2805 | Yes |
| 12 | CTSC |  |  | 531 | -0.470 | -0.2255 | Yes |
| 13 | PLAT |  |  | 556 | -0.538 | -0.1802 | Yes |
| 14 | CLU |  |  | 581 | -0.690 | -0.1105 | Yes |
| 15 | MT3 |  |  | 591 | -0.837 | 0.0086 | Yes |
Table: GSEA details [plain text format]

  

Fig 2: HALLMARK\_COMPLEMENT: Random ES distribution      
 Gene set null distribution of ES for **HALLMARK\_COMPLEMENT**
